# Assessing the Prevalence and Determinants of Exposure-Influenced HIV Testing among a Sample of Pre- and Post-Exposure Prophylaxis-Naïve Young Men Who Have Sex with Men in the United States

**DOI:** 10.3390/tropicalmed7080146

**Published:** 2022-07-26

**Authors:** Yu Liu, Mary Hawkins, Amna Osman, Chen Zhang

**Affiliations:** 1 Division of Epidemiology, Department of Public Health Sciences, University of Rochester Medical Center, 256 Crittenden Blvd., Ste. 3305, Rochester, NY 14642, USA; 2 Nashville Council on AIDS, Resources, Education and Support (CARES), Nashville, TN 37204, USA; mhawkins@nashvillecares.org (M.H.); aosman@nashvillecares.org (A.O.); 3 School of Nursing, University of Rochester, Rochester, NY 14642, USA; chen_zhang@urmc.rochester.edu

**Keywords:** HIV testing, men who have sex with men, psychosocial, sexual risk, pre-exposure prophylaxis, United States

## Abstract

Self-initiated Human Immunodeficiency Virus (HIV) testing after potential sexual exposure to HIV (i.e., exposure-influenced HIV testing) has high utility in detecting individuals with the highest probabilities of HIV seroconversion. We conducted a cross-sectional study among a sample of sexually active, pre/post-exposure prophylaxis (PrEP/PEP)-naïve young men who have sex with men (YMSM) in two US cities to assess the determinants (e.g., demographic, psychosocial, sexual, substance use, and HIV prevention characteristics) of exposure-influenced HIV testing (*never/rarely* vs. *mostly/always*) in their lifetime. Of 261 YMSM, only 26.5% reported *mostly/always* seeking exposure-influenced prior to the study. Multivariable analyses showed that younger age, sexual orientation non-disclosure, perceived HIV stigma, internalized homophobia, lower general resilience, and lower social support were associated with a lower likelihood of *mostly/always* seeking exposure-influenced HIV testing. YMSM who *never/rarely* sought exposure-influenced HIV testing were more likely to use recreational drugs before sex, binge alcohol, and have group sex; while less likely to be aware of PrEP, test for sexually transmitted infections, or use condoms compared to those *mostly/always* seeking exposure-influenced HIV testing. Exposure-influenced HIV testing is suboptimal among YMSM with elevated risk for HIV. Our findings provide important implications for designing targeted interventions to promote exposure-influenced HIV testing among high-risk YMSM.

## 1. Introduction

Young men who have sex with men (YMSM) aged 18–35 years accounted for more than 50% of all new Human Immunodeficiency Virus (HIV) infections in the United States (US) in 2019 [1,2]. HIV testing is crucial to the implementation of effective HIV treatment and intervention strategies, such as “test-and-treat” for newly HIV-diagnosed individuals and pre-exposure prophylaxis (PrEP) linkage for those with high risk for HIV [3,4,5,6]. Without timely HIV screening, the undiagnosed infections may further exacerbate HIV transmissions among YMSM via condomless sex and result in poor HIV care continuum outcomes among HIV-positive YMSM who are not effectively linked to care or treated [7,8].

The US Centers for Disease Control and Prevention (CDC) recommends annual HIV testing, with some HIV prevention experts suggesting more frequent HIV testing (e.g., every 3–6 months) for sexually active YMSM irrespective of reported risk [9,10,11,12]. Despite the expansion of various HIV testing programs for YMSM, HIV testing remains suboptimal among this subgroup [13,14]. One meta-analysis showed that only 60% of 83,286 racially diverse YMSM recruited from the internet had tested for HIV in the past 12 months [15]. Another meta-analysis among 18,253 young black men who have sex with men (MSM) found only 42% reporting HIV testing every 3–6 months in the past year [16]. A national surveillance study estimated that 20% of 10,104 MSM from 23 US cities were living with undiagnosed HIV [17].

Although numerous studies have assessed the prevalence and determinants of HIV testing among YMSM in the US [18,19,20,21,22], no study has been conducted to explore this topic by differentiating the contexts in which HIV testing is initiated. For instance, whether HIV testing is passive due to the requirement of research participation, PrEP candidacy evaluation, or financial incentives (i.e., passive HIV testing); more importantly, whether the HIV testing decision is actively made due to the influence of recent sexual exposure to HIV (i.e., exposure-influenced HIV testing). Passive and active (i.e., exposure-influenced) HIV testing can have substantially varying utilities for HIV detection because the latter is more likely to capture individuals with the highest probabilities of HIV seroconversion, particularly for those who are PrEP/PEP-naïve [23,24,25,26]. While engaging YMSM in passive HIV testing regardless of sexual risks is a vital strategy for detecting new HIV infections, strategies and resources should also be prioritized to educate and promote exposure-influenced HIV testing among high-risk YMSM, which is essential to bolster US CDC′s overarching HIV testing guidelines and objectives.

The primary purpose of this study is to bridge the knowledge gap of exposure-influenced HIV testing among YMSM in the US. Therefore, we conducted an epidemiologic study among a sample of non-PrEP/PEP using, high-risk YMSM recruited from two US cities with the following objectives: (1) to assess the prevalence of exposure-influenced HIV testing; (2) to explore relevant demographic, psychosocial and behavioral correlates; (3) to evaluate the self-report intention of future exposure-influenced HIV testing uptake; and (4) to understand the barriers/facilitators of seeking exposure-influenced HIV testing. Evidence from the present study may help inform the design and implementation of future targeted interventions to promote exposure-influenced HIV testing among YMSM with elevated risk for HIV.

## 2. Materials and Methods

### 2.1. Study Design and Participants

A cross-sectional (May 2019–May 2020) study was conducted among YMSM living in two US cities (e.g., Nashville, Tennessee, and Buffalo, New York). Details of the materials and methods have been documented elsewhere [27,28,29]. In short, we partnered with the Nashville Council on AIDS, Resources, Education, and Support (Nashville CARES) to recruit participants via flier distribution, peer referral, community outreach, and social media advertisement. In Buffalo, we collaborated with Evergreen Health Services to recruit participants from community HIV clinics by approaching potential participants during their walk-ins. Participants were eligible if they met the following criteria: 18–35 years of age, residents of Nashville or Buffalo, cis-gender man, HIV-negative or status-unknown, had at least one episode of condomless anal sex with a man in the past 12 months, and can read/understand English.

### 2.2. Data collection and Measures

A 40-min internet-assisted questionnaire survey was administered to the consented YMSM. Participants were allowed to complete the questionnaire onsite in a private, tablet-equipped room or have the research staff send a secure survey link to them to complete the survey elsewhere. We collected data on: (1) sociodemographic characteristics (e.g., age, race, employment status, educational attainment, annual personal income, sexual orientation, health insurance); (2) psychosocial factors (e.g., HIV stigma, internalized homonegativity, subjective loneliness, perceived social support, resilience, food security, and housing stability) [29]; (3) risky behaviors (e.g., venues for finding sex partners, patterns of tobacco, recreational drug use, alcohol misuse patterns, condomless insertive/receptive anal sex (CIAS/CRAS), oral/group sex with men, and history of sexually transmitted infections (STI)); and (4) HIV prevention indicators (e.g., history of HIV/STI testing, the intention of future exposure-influenced HIV testing (high/moderate vs. low/no), perceived HIV testing barriers/facilitators, PrEP awareness, and condom use self-efficacy). The primary outcome variable was the tendency of exposure-influenced HIV testing uptake. We asked the participants how often in their lifetime had they purposively sought HIV testing after engagement in one or more (i.e., consecutive) types/episodes of the following risk behaviors: (1) CIAS/CRAS with a casual sex partner, (2) CIAS/CRAS with a known HIV-positive partner (i.e., HIV-positivity disclosed by the partners regardless of the knowledge of viral suppression status), (3) condomless oral sex with a causal or known HIV-positive partner, (4) group sex, and (5) alcohol/drug use before sex. Responses were initially recorded as *never, rarely, mostly, and always*, and further dichotomized as *never/rarely (i.e., infrequent exposure-influenced testers)* vs. *mostly/always (i.e., frequent exposure-influenced testers)* to increase the sample size in each response category.

### 2.3. Statistical Analyses

Descriptive analyses (frequency distribution or central tendency) were conducted for all variables. Bivariate analyses (e.g., chi-square/Fisher′s exact tests and Mann–Whitney U tests) were conducted to compare the differences in demographics, psychosocial determinants, risk behaviors, and selected HIV prevention indicators between frequent exposure-influenced testers (*mostly/always*) and infrequent exposure-influenced testers (*never/rarely*). To assess the independent associations of demographic and psychosocial predictors of exposure-influenced HIV testing, variables at the significance of *p* < 0.2 were selected to fit a log-linked Poisson regression model with robust standard errors to estimate the adjusted prevalence ratio (aPR) and 95% confidence interval (CI), followed by a backward elimination procedure to retain factors at the significance of *p* < 0.05 in the final multivariable model. All statistical analyses were conducted using Stata 14.0^TM^ (StataCorp LP, College Station, TX, USA).

### 2.4. Ethical Considerations

Informed consent was obtained from all participants prior to enrollment. The study procedure and protocol were reviewed and approved by the Institutional Review Boards at the University of Rochester and the University at Buffalo.

## 3. Results

### 3.1. Participant Characteristics

A total of 415 eligible YMSM agreed to participate in the study, and 347 enrolled and completed the survey in full (response rate: 83.6%). Of the 347 YMSM, 261 self-reported as non-PrEP/PEP users and were included for analyses in the current study. The median age of the analytical sample (N = 261) was 25 years (interquartile range: 22–27 years), with 68.6% being Black, 66.3% being employed, 32.9% having college and above education, 48.7% reporting < USD 20 k annual personal income, 79.3% being health insured, and 71.6% reporting as gay/homosexual (Table 1).

### 3.2. Demographic and Psychosocial Correlates of Exposure-Influenced HIV Testing

We found only 26.5% (n = 69) reported *mostly/always* seeking exposure-influenced HIV testing (i.e., as frequent exposure-influenced testers). Compared to frequent exposure-influenced testers, infrequent exposure-influenced testers (i.e., those who *never/rarely* tested) were more likely (*p* < 0.05) to be younger, younger when they first had CIAS/CRAS with men, not disclose their sexual orientation to healthcare providers, use internet/website to find sex partners, report a higher level of internalized homophobia, and report a lower level of perceived social support. Multivariable analyses showed that older age (25–35 vs. 18–24 years; aPR = 2.35; 95% CI: 1.12–4.97), sexual orientation disclosure to healthcare providers (yes vs. no; aPR = 2.63; 95% CI: 1.29–5.36), a higher level of perceived social support (one unit increase in the perceived social support score; PR = 1.02; 95% CI: 1.01–1.03), and a higher level of general resilience (one unit increase in the general resilience score; PR = 1.03; 95% CI: 1.01–1.07) were associated with a higher likelihood of *mostly/always* seeking exposure-influenced HIV testing; while YMSM who had moderate/high HIV risk perception (vs. no/low risk perception; aPR = 0.29; 95% CI: 0.13–0.67), a higher level of perceived HIV stigma (one unit increase in the perceived HIV stigma score; aPR = 0.89; 95% CI: 0.84–0.96), and a higher level of internalized homophobia (one unit increase in the internalized homophobia score; aPR = 0.91; 95% CI: 0.82–0.98) were more likely to *never/rarely* seek exposure-influenced HIV testing in their lifetime (Table 1).

### 3.3. Comparison of Behavioral Characteristics

Compared to frequent exposure-influenced testers (Table 2), infrequent testers were more likely to report recent (i.e., past 6-month) recreational drug use before sex (40.6% vs. 30.4%; *p* = 0.048), recent alcohol binge drinking (78.6% vs. 53.6%; *p* < 0.001), and recent engagement in group sex (27.1% vs. 14.5%; *p* = 0.038). On the contrary, frequent exposure-influenced testers were more likely to have ever tested for STIs (95.6% vs. 80.7%; *p* = 0.003), have a history of STIs (39.1% vs. 28.7%; *p* = 0.049), have more lifetime HIV testing (median times: 10 vs. 3; *p* < 0.001), have a higher level of condom use self-efficacy (median score: 33 vs. 29; *p* = 0.013), and be aware of PrEP (87.0% vs. 70.3%; *p* = 0.006) compared to their infrequent tester counterparts.

### 3.4. The Intention of Seeking Future Exposure-Influenced HIV Testing

**Figure 1:**
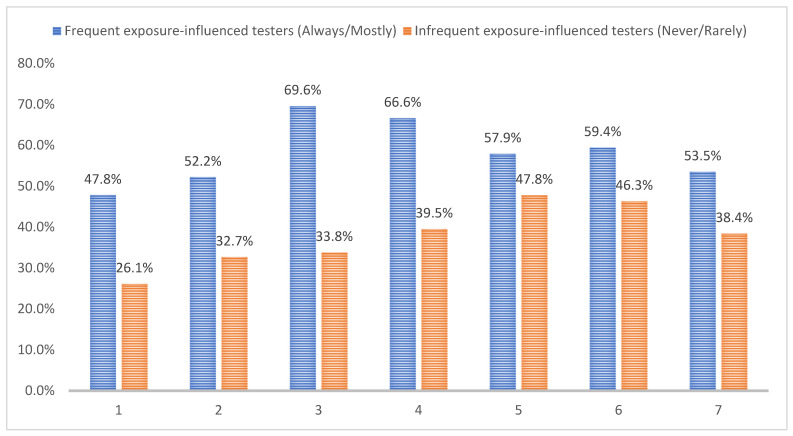
Proportions of YMSM reporting high/moderate (vs. low/no) intention of seeking future exposure-influenced HIV testing by various risky sex scenarios among previously frequent (n = 69) and infrequent (n = 192) exposure-influenced testers. Note: 1—condomless insertive anal sex with a regular male partner; 2—condomless receptive anal sex with a regular male partner; 3—condomless insertive anal sex with a casual male partner; 4—condomless receptive anal sex with a casual male partner; 5—condomless insertive/receptive anal sex with a known HIV-positive male partner; 6—group sex with men; 7—condomless oral sex with men. Statistical significance (α = 0.05) of comparing the proportion of previously frequent vs. infrequent risk-motivated HIV testing YMSM who reported high/moderate confidence in seeking future risk-motivated HIV testing by various risky sex scenarios: *p* < 0.01 (1, 2, 3 and 4); *p* < 0.05 (6, 7).

Figure 1 shows the proportions of YMSM reporting high/moderate intention in initiating exposure-influenced HIV testing in the future. Overall, frequent exposure-influenced testers were more likely (*p* < 0.05) to report high/moderate intention to seek future HIV testing after engaging in all hypothetical risky sex scenarios. Specifically, among previous frequent exposure-influenced testers, having CIAS with a casual male partner represents the most likely scenario to seek future exposure-influenced HIV testing (69.6%), followed by having CRAS with men (66.6%) and group sex (59.4%). Among infrequent exposure-influenced testers, having CIAS/CRAS with HIV-positive partners was associated with the highest proportion (47.8%) of YMSM reporting high/moderate intention to test for HIV, followed by having group sex (46.3%) and CRAS with a casual male partner (39.5%). Among both frequent and infrequent exposure-influenced testers, having CIAS with a regular male partner was associated with the lowest proportion (47.8% and 26.1%, respectively) reporting high/moderate intention to seek subsequent HIV testing.

### 3.5. Barriers and Facilitators of Seeking Exposure-Influenced HIV Testing

**Figure 2:**
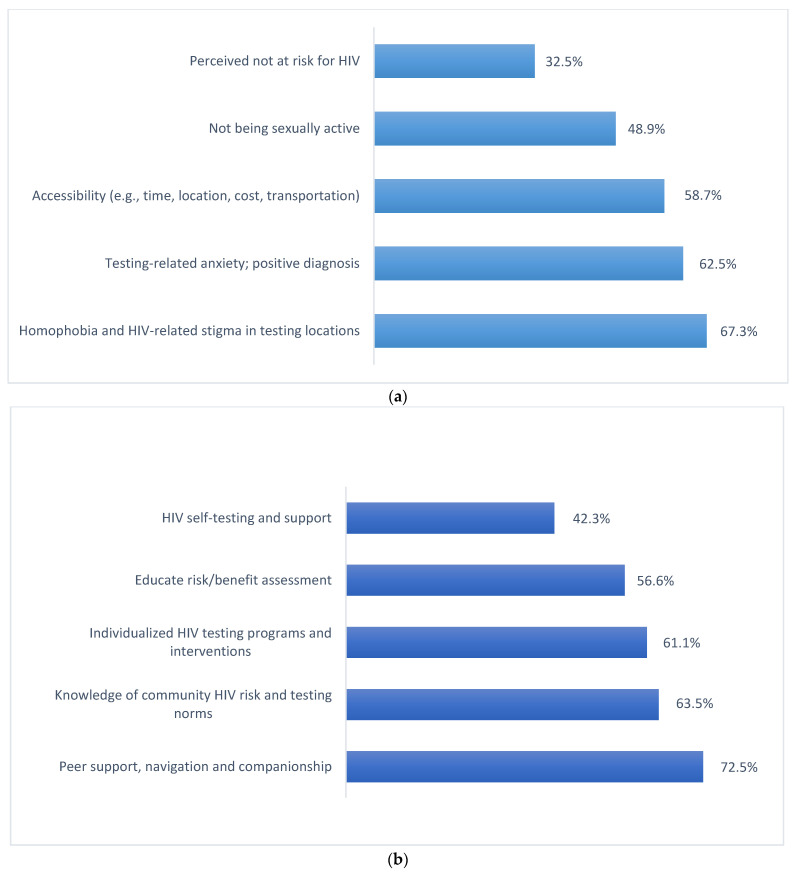
Summary of top identified (**a**) barriers to and (**b**) facilitators of enhancing exposure-influenced HIV testing among a sample of PrEP/PEP-naïve YMSM in two US urban areas (N = 261).

Figure 2 presents the top-five cited barriers to and facilitators of seeking exposure-influenced HIV testing. Homophobia and HIV-related stigma represent the most cited barrier (67.3%), followed by testing-related anxiety/fear (62.5%), HIV testing accessibility (58.7%), sexual inactivity (48.9%), and low HIV risk perception (32.5%). Among all the facilitators, participants cited peer support/navigation as the most important driver (72.5%), followed by HIV testing descriptive norms in the community (63.5%), individualized HIV testing promotion intervention (61.1%), education of risk/benefit of HIV testing (56.6%), and the support of using HIV self-testing (42.3%).

## 4. Discussion

HIV testing uptake represents a critical step to reducing onward HIV transmissions through undiagnosed infections and enhancing the continuum of HIV care outcomes (e.g., rapid linkage-to-care, HIV treatment initiation, and viral suppression) for YMSM at high risk for HIV [4,30,31]. With the increase in the availability and accessibility of HIV testing services, we have observed a steadily increasing trend of HIV testing uptake among YMSM of all racial/ethnic groups in recent years in the US [1]. Our study found that 91.5%, 76.3%, and 68.2% of the participants reported HIV testing in their lifetime, the past 12 months, and the past 6 months, respectively (data not shown in tables). This finding was consistent with a nationally representative YMSM study—88.2% tested in their lifetime, 67.4% tested in the past 12 months, and 63.4% tested in the past 6 months, respectively) [16]. In the meantime, our results were in line with findings from numerous studies showing a decreasing HIV testing prevalence from lifetime to more recent time windows (e.g., past 3–6 months), highlighting the need for continuous efforts to strengthen recent and frequent HIV testing among YMSM in the US [7,15,16,32,33].

To our best knowledge, this is the first epidemiologic study to explore the prevalence, psychosocial/behavioral correlates, and barriers/facilitators of exposure-influenced HIV testing among a sample of non-PrEP/PEP using YMSM in the US. The current study found a substantially low proportion (26.5%) of YMSM reporting *mostly/always* seeking exposure-influenced HIV testing. The low prevalence of exposure-influenced HIV testing has several important implications. From the standpoint of HIV prevention, exposure-influenced HIV testing must be strengthened because it represents a vital HIV testing mechanism to cost-effectively detect individuals with high HIV risks (i.e., sexually active, non-PrEP/PEP using YMSM) for timely linkage to HIV prevention or care. From the perspective of research design and result interpretation, HIV prevention scientists should avoid being over-optimistic when interpreting the seemingly high HIV testing prevalence in an observational study. The high HIV testing prevalence can likely be “inflated” by individuals who frequently test for HIV but are not at high risk for HIV (e.g., the “worried well,” consistent PrEP users, low-risk individuals who frequently participate in HIV research) [23]. Researchers should carefully craft relevant questionnaires to specify the contexts in which an HIV testing is initiated and use caution when calculating relevant HIV testing prevalence across various participants (e.g., PrEP users vs. non-users).

We found that MSM of younger ages were less likely to actively seek HIV testing after sexual exposure to HIV, which is consistent with previous studies showing that YMSM (vs. older MSM) were less representative in HIV prevention uptake (e.g., HIV testing, PrEP) [34,35,36]. Studies have also shown that the lower likelihood of HIV testing among YMSM could be partially attributable to low HIV risk perception, less experience navigating various HIV prevention services, and higher substance use [37,38,39]. These explanations are also supported by our previous studies with the same study participants [27,28,29]. Surprisingly, we found that HIV risk perception was negatively associated with exposure-influenced HIV testing uptake. This finding contradicts previous studies showing HIV risk perception as a strong predictor of frequent HIV testing uptake among YMSM [40,41,42]. We suspect that the association between risk perception and HIV prevention uptake may be mediated by various psychosocial factors (e.g., anxiety, stress, homophobia and stigma), which are likely to erode the psychological capacity of HIV testing decision making among some YMSM, despite the perception of higher risk for HIV [27,43,44]. In particular, HIV/gay-related stigma may significantly exacerbate HIV prevention uptake among YMSM [45,46]. In our study, perceived HIV/gay-related discrimination and stigma at HIV testing sites were cited by 67% of the study participants as the major barrier to seeking exposure-influenced HIV testing. Therefore, hospitals/clinics and local HIV prevention centers must build a more affirmative, holistic, and safe HIV testing environment to help alleviate concerns of intersectional HIV/gay stigma, HIV testing-related anxiety, and facilitate sexual orientation disclosure to healthcare providers—all critical factors to promote HIV testing among YMSM [47,48]. Last, we found that individualized support across various physical, normative, psychological, and environmental domains to promote resiliency is cited as an essential facilitator to promote HIV testing uptake among YMSM. Future studies should further explore sources across multiple socio-ecological levels to guide the design and implementation of multidimensional social support and resilience-based HIV prevention programs to help address various psychosocial vulnerabilities and promote exposure-influenced HIV testing uptake among YMSM [49].

In line with findings from previous studies conducted among MSM in Tennessee and New York [50,51,52,53,54,55], a high prevalence of substance use (e.g., alcohol, recreational drugs, and tobacco) and condomless anal sex (e.g., CIAS and CRAS) was observed among our study participants recruited in these areas. We also found comparable sexual risk portfolios between participants who *never/rarely* and *mostly/always* sought exposure-influenced HIV testing in the study, with those *never/rarely* seeking exposure-influenced HIV testing showing a higher prevalence in alcohol binging, recreational drug use before sex, and group sex—all established risk factors for HIV acquisition. The mechanism of the association between substance use-induced HIV risks, and the lower propensity in seeking HIV testing can be complex in different contexts. In this study, infrequent exposure-influenced HIV testers were more likely to report a lower level of resilience and perceived social support and a higher level of subjective loneliness. Evidence suggests these factors are important predictors of substance use, subsequent sexual disinhibition, and impaired HIV prevention decision making [34,56,57]. We found that YMSM who engaged more frequently in previous HIV/STD testing were more likely to seek exposure-influenced HIV testing. We also found that frequent exposure-influenced HIV testers reported higher intention to seek future HIV testing than their infrequent tester counterparts. These findings are well supported by the self-efficacy theory, in which mastery experience (i.e., previous experience may boost confidence in future events) represents a key theoretical construct in behavioral prediction [58]. Future theory-based interventions should capitalize on personal relevance and enhance intrinsic motivation for HIV prevention uptake among YMSM.

The major strength of this study includes the first assessment of exposure-influenced HIV testing and relevant intervenable correlates among a community-based sample consisting of a large presence of racial minority men (e.g., young black MSM) at high risk for HIV. There are also limitations to the study. First, the generalizability of the study findings is limited due to the small sample size of participants recruited from two US cities. Second, the study′s cross-sectional nature may not reveal the temporal relationship of the observed associations; therefore, the causality of our findings should be interpreted with caution. Third, self-report data might be subjected to recall bias and social desirability, resulting in underreporting of sensitive questions (e.g., substance use, sexual history). However, we expected that the impact of such bias was minimal based on how we collected the data (e.g., anonymous and self-administered in a private location/facility). Fourth, the outcome assessment may be limited by only ascertaining the lifetime tendency (e.g., never, rarely, mostly, always) of exposure-influenced HIV testing uptake rather than the frequency (e.g., count) or event-level testing (i.e., asking whether or not an HIV testing was initiated after engagement in a specific risky sexual event). Despite this limitation, we considered that the tendency assessment would be less affected by recall bias than asking the participants to recall any specific count/events. Last, due to the small sample size, we were limited to performing various stratified analyses (e.g., sexual history, race, number of lifetime sexual partners) to further explore the patterns of exposure-influenced HIV testing within each YMSM subgroup.

## 5. Conclusions

HIV testing represents an essential gateway for PrEP initiation and HIV care engagement among individuals at elevated risk for HIV. Promoting active, self-initiated HIV testing for high-risk YMSM (e.g., PrEP/PEP-naïve YMSM who frequently engage in risky sexual episodes) is particularly important, as it allows those with the highest probability of HIV seroconversion to be cost-effectively detected and linked to care for preventing further HIV transmission among the MSM community. However, we found that this type of active HIV testing uptake (i.e., exposure-influenced HIV testing) was low among high-risk YMSM. Our findings shed important light on targeted intervention opportunities to help strengthen exposure-influenced HIV testing among YMSM in the US.

## Figures and Tables

**Table 1:** Demographic and psychosocial correlates of exposure-influenced HIV testing among a sample of PrEP/PEP-naïve young men who have sex with men recruited from two US urban areas (N = 261).

Characteristics		Exposure-Influenced HIV Testing ^a^					
	Total (N = 261)	*Never/Rarely* (N = 192)		*Always/Mostly* (N = 69)		*p*-Value	aPR (95% CI) ^b^
	n (%) or Median (IQR)	n (%) or Median (IQR)		n (%) or Median (IQR)			
**Age (years)**						0.005	
18–24	123 (47.5)	101	(52.6)	23	(33.3)		Reference
25–35	137 (52.5)	91	(47.4)	46	(66.7)		2.35 (1.12–4.97)
**Age of first condomless anal sex (years)**						0.049	
<18	132 (50.6)	104	(54.2)	28	(40.6)		
≥18	129 (49.4)	88	(45.8)	41	(59.4)		
**Race**						0.583	
Non-Hispanic white	61 (23.4)	48	(25.0)	13	(18.8)		
Non-Hispanic black	179 (68.6)	129	(67.2)	50	(72.5)		
Other ^†^	21 (8.0)	15	(7.8)	6	(8.7)		
**Employment**						0.077	
Currently employed	173 (66.3)	124	(64.6)	49	(71.0)		
Currently unemployed	43 (16.5)	29	(15.1)	14	(20.3)		
Currently a student	45 (17.2)	39	(20.3)	6	(8.7)		
**Education**						0.141	
High school or lower	65 (24.9)	52	(27.1)	13	(18.9)		
Some college	110 (42.2)	83	(43.2)	27	(39.1)		
College and above	86 (32.9)	57	(29.7)	29	(42.0)		
**Annual personal income (US dollars)**						0.424	
<USD 20,000	127 (48.7)	98	(51.0)	29	(42.0)		
USD 20,000–40,000	85 (32.6)	59	(30.7)	26	(37.7)		
>USD 40,000	49 (18.7)	35	(18.3)	14	(20.3)		
**Sexual orientation**						0.117	
Homosexual/gay	187 (71.6)	131	(68.2)	56	(81.2)		
Heterosexual	32 (12.3)	27	(14.1)	5	(7.2)		
Bisexual	42 (16.1)	34	(17.7)	8	(11.6)		
**Sexual orientation disclosure to healthcare professionals**						0.006	
No	75 (28.7)	64	(33.3)	11	(15.9)		Reference
Yes	186 (71.3)	128	(66.7)	58	(84.1)		2.63 (1.29–5.36)
**Venues for finding sex partners**						0.045	
Gay-frequented venues (bars, clubs, etc.)	47 (18.0)	35	(18.2)	12	(17.4)		
Internet (Facebook, Reddit, etc.)	43 (16.5)	38	(19.8)	5	(7.2)		
Geosocial networking app (Grindr, etc.)	171 (65.5)	119	(62.0)	52	(75.4)		
**Perception of HIV risk**						0.064	
No/low risk	198 (75.9)	140	(72.9)	58	(84.1)		Reference
Moderate/high risk	63 (24.1)	52	(27.1)	11	(15.9)		0.29 (0.13,0.67)
**Health insurance**						0.924	
No	54 (20.7)	40	(20.8)	14	(20.3)		
Yes	207 (79.3)	152	(79.2)	55	(79.7)		
**Housing stability (1–10), median (IQR)**	9 (6–10)	9	(5–10)	9	(7–10)	0.617	
**Food security (0–6), median (IQR)**	1 (0–4)	1	(0–4)	0	(0–2)	0.098	
**Perceived HIV stigma (12–48), median (IQR)**	30 (24–36)	32	(29–36)	29	(24–32)	0.031	0.89 (0.84–0.96)
**Internalized homophobia (4–20), median (IQR)**	5 (4–12)	6	(4–12)	4	(4–8)	0.038	0.91 (0.82–0.98)
**Perceived social support (8–32), median (IQR)**	74 (57–90)	73	(57–87)	76	(62–95)	0.042	1.02 (1.01–1.03)
**Subjective loneliness (19–95), median (IQR)**	19 (15–23)	19	(16–23)	18	(14–23)	0.472	
**General resilience (0–40), median (IQR)**	29 (23–36)	28	(22–35)	30	(26–36)	0.061	1.03 (1.01–1.07)

Note: aPR, adjusted prevalence ratio; CI, confidence interval; ^†^ Including Hispanic/Latino, Asian, and Refuse to answer. ^a^ Self-initiated HIV testing after engagement in one or more episodes of the following risk behaviors: condomless anal sex (CAS) with a casual sex partner, CAS with a known HIV-positive partner, condomless oral sex with a causal and/or HIV-positive partner, group sex, and/or alcohol/drug intoxication before sex. ^b^ Adjusted for variables with a *p*-value < 0.2 from bivariate analyses and only retaining/reporting the significant correlates (*p* < 0.05) in the table after backward selection procedure.

**Table 2:** Comparing the prevalence of substance use, sexual behaviors and HIV prevention indicators by exposure-influenced HIV testing among a sample of PrEP/PEP-naïve young men who have sex with men recruited from two US urban areas (N = 261).

Characteristics		Exposure-Influenced HIV Testing ^a^				
	Total (N = 261)	*Never/Rarely* (N = 192)		*Always/Mostly* (N = 69)		*p*-Value
	n (%) or Median (IQR)	n (%) or Median (IQR)		n (%) or Median (IQR)		
**Ever used any tobacco product ^‡.^**						0.534
No	61 (23.4)	43	(22.4)	18	(26.1)	
Yes	200 (76.6)	149	(77.6)	51	(73.9)	
**Ever used any recreational drug ^†^**						0.947
No	56 (21.5)	41	(21.3)	15	(21.7)	
Yes	205 (78.5)	151	(78.7)	54	(78.3)	
**Recreational drug use before sex in the past 6 months**						**0.048**
No	162 (62.1)	114	(59.4)	48	(69.6)	
Yes	99 (37.9)	78	(40.6)	21	(30.4)	
**Ever drank alcohol**						0.478
No	41 (15.7)	32	(16.7)	9	(13.0)	
Yes	220 (84.3)	160	(83.3)	60	(87.0)	
**Hazardous alcohol drinking in the past 6 months**						0.439
No (AUDIT-C < 4)	154 (59.0)	116	(60.4)	38	(55.1)	
Yes (AUDIT-C ≥ 4)	107 (41.0)	76	(39.6)	31	(44.9)	
**Binge drinking in the past 6 months ^※^**						**0.002**
No	73 (28.0)	41	(21.4)	32	(46.4)	
Yes	188 (72.0)	151	(78.6)	37	(53.6)	
**Alcohol use before sex**						0.259
No	121 (46.4)	85	(44.3)	36	(52.2)	
Yes	140 (53.6)	107	(55.7)	33	(47.8)	
**Lifetime male sex partner**						0.863
<10	137 (53.5)	100	(53.2)	37	(54.4)	
≥10	119 (46.5)	88	(46.8)	31	(45.6)	
**Had group sex in the past 6 months**						**0.036**
No	194 (76.4)	135	(72.9)	59	(85.5)	
Yes	60 (23.6)	50	(27.1)	10	(14.5)	
**Had condomless insertive anal sex with men in the past 6 months**						0.553
No	114 (45.1)	85	(46.2)	29	(42.0)	
Yes	139 (54.9)	99	(53.8)	40	(58.0)	
**Had condomless receptive anal sex with men in the past 6 months**						0.404
No	125 (49.2)	94	(50.8)	31	(44.9)	
Yes	129 (50.8)	91	(49.2)	38	(55.1)	
**Had anal and/or oral sex with known HIV-positive partners in the past 6 months**						0.321
No	200 (81.3)	142	(79.8)	58	(85.3)	
Yes	46 (18.7)	36	(20.2)	10	(14.7)	
**Ever had sexually transmitted disease testing**						**0.003**
No	40 (15.3)	37	(19.3)	3	(4.4)	
Yes	221 (84.7)	155	(80.7)	66	(95.6)	
**History of sexually transmitted infections ^§^**						**0.049**
No	179 (68.6)	137	(71.3)	42	(60.9)	
Yes	82 (31.4)	55	(28.7)	27	(39.1)	
**HIV pre-exposure awareness**						**0.006**
No	66 (25.3)	57	(29.7)	9	(13.0)	
Yes	195 (74.7)	135	(70.3)	60	(87.0)	
**Lifetime number of HIV testing, median (IQR)**	4 (2–8)	3	(1–6)	10	(5–17)	**<0.001**
**Condom use self-efficacy (5–35), median (IQR)**	30 (26–34)	29	(25–34)	33	(28–35)	**0.013**

Note: AUDIT-C, Alcohol Use Disorders Identification Test—Consumption (score range: 0–12); bold indicates statistical significance (*p* < 0.05). ^‡^ Including intake or smoking (even a puff) of the following products: Regular cigarette, E-cigarette, Bidi, Cigar, Hookah, Pipe, Dip, Chewing tobacco, Dissolvable, Snuff, or Snus. ^†^ Recreational drug use: self-report intake of rush poppers (alkyl nitrites), crystal meth (methamphetamine), marihuana, hallucinogens (ketamine, LSD, PCP, etc.), cocaine, heroin or other opioids, Magu (a mixture of methamphetamine and caffeine), opium, triazolam tablets (benzodiazepines) or ecstasy (3,4-methylenedioxymethamphetamine, MDMA). **^※^** Binge drinking is defined as having six or more standard drinks (i.e., 12 ounces (one can) of beer (5% alcohol), 6 ounces (one glass) of wine (12% alcohol), 1.5 ounces (one shot) of liquor (40% alcohol)) during a drinking occasion. **^§^** Including previous diagnosis of one or more of the following sexually transmitted infections: hepatitis B/C, syphilis, gonorrhea, chlamydia, herpes simplex virus (HSV), human papillomavirus (HPV), and/or trichomoniasis. ^a^ Self-initiated HIV testing after engagement in one or more episodes of the following risk behaviors: condomless anal sex (CAS) with a casual sex partner, anal sex with a known HIV-positive partner, condomless oral sex with a causal and/or known HIV-positive partner, group sex, and/or alcohol/drug intoxication before sex.

## Data Availability

Limited de-identified raw data are available from the corresponding author upon reasonable request.
